# Notes from the Field: Lead Poisoning in an Infant Associated with a Metal Bracelet — Connecticut, 2016

**DOI:** 10.15585/mmwr.mm6634a6

**Published:** 2017-09-01

**Authors:** Patricia Garcia, Jennifer Haile

**Affiliations:** 1 Hartford Regional Lead Treatment Center, Connecticut Children’s Medical Center.

In September 2016, routine screening of a female infant aged 9 months in Manchester, Connecticut, showed normocytic anemia and a blood lead level of 41 *μ*g/dL (levels exceeding 5 *μ*g/dL are abnormal) (*1*). The child was cared for only in the home, which was built in 1926. Epidemiologic investigation identified two interior window wells with peeling lead-based paint; however, the health department concluded that the windows were unlikely the source of exposure, given the lack of accessibility to these areas by the child. The child’s three siblings, ranging in age from 3–5 years, had blood lead levels of <3 *μ*g/dL.

The parents reported that the child intermittently wore a handmade “homeopathic magnetic hematite healing bracelet” that they had purchased from an artisan at a local fair (Figure). The child wore the bracelet for teething related discomfort and was sometimes noted to chew on it. Small spacer beads from the bracelet tested at the Manchester Health Department were positive for lead (17,000 ppm). No identifying marks indicating metal content or manufacturer were found on the bead. The vendor records were not available, and the bracelet maker could not be located.

**FIGURE:**
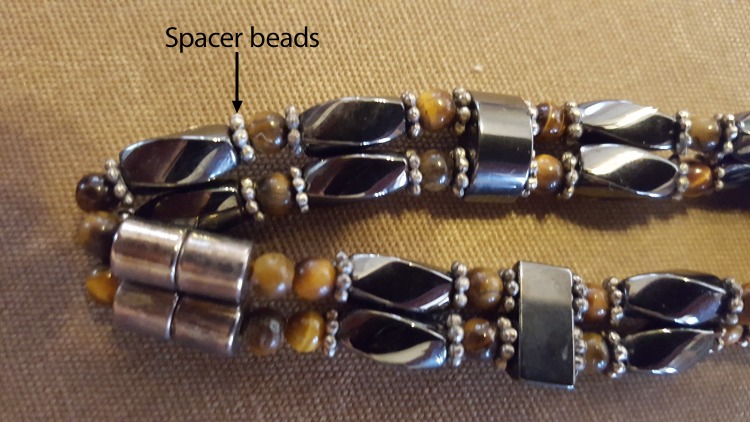
Bracelet with spacer beads containing lead, resulting in lead poisoning of an infant — Connecticut, 2016 **Photo/**Kimberly Dubanoski, Manchester Health Department, Connecticut

Lead poisoning occurs primarily through oral ingestion of lead containing products. Lead paint, dust, and contaminated soil are the most common sources of lead exposure in children; however, nonpaint sources are often identified in acute poisonings (*1*,*2*). Cases of severe lead poisoning and death were linked to lead-containing charms and jewelry marketed to children in 2003 (*3*) and 2006 (*4*), resulting in large-scale recalls. In 2010, the Consumer Product Commission set the limit of lead content in items manufactured and marketed for children at 100 ppm.* This standard results in numerous recalls of children’s jewelry each year^†^^,^^§^ but does not apply to items that are not intended for use by or in children’s products.

Clinicians should be aware of the potential for lead poisoning in children who have ingested or mouthed any metal objects, especially jewelry. Caregivers should be made aware of the risks for lead poisoning resulting from children wearing or handling handmade or adult metal jewelry, even if items are manufactured or purchased in the United States, because infants have natural mouthing behaviors; these items can also pose a choking hazard for small children (*5*). Cases of lead poisoning in these situations should be reported immediately to local public health departments so that timely investigation can be conducted and the source eliminated to prevent further cases of poisoning.

## Footnotes

* Consumer Product Safety Improvement Act of 2008. https://www.cpsc.gov/s3fs-public/pdfs/blk_media_cpsia.pdf.

† Consumer Product Safety Commission. Mars retail group recalls M&M branded jewelry due to violation of lead standard. 2016. https://www.cpsc.gov/Recalls/2016/mars-retail-group-recalls-mms-branded-jewelry.

§ Consumer Product Safety Commission. Things Remembered recalls children’s jewelry due to violation of lead standard. 2016. https://www.cpsc.gov/Recalls/2016/things-remembered-recalls-childrens-jewelry.

## References

[1] CDC. Blood lead levels in children aged 1–5 years—United States, 1999–2010. MMWR Morb Mortal Wkly Rep 2013;62:245–8. 23552225 PMC4605011

[2] Gorospe EC, Gerstenberger SL. Atypical sources of childhood lead poisoning in the United States: a systematic review from 1966–2006. Clin Toxicol (Phila) 2008;46:728–37. 10.1080/15563650701481862 18608287

[3] CDC. Lead poisoning from ingestion of a toy necklace—Oregon, 2003. MMWR Morb Mortal Wkly Rep 2004;53:509–11. 15201845

[4] CDC. Death of a child after ingestion of a metallic charm—Minnesota, 2006. MMWR Morb Mortal Wkly Rep 2006;55:340–1. 16572103

[5] Committee on Injury, Violence, and Poison Prevention. Prevention of choking among children. Pediatrics 2010;125:601–7. 10.1542/peds.2009-2862 20176668

