# *Erratum: *Vol. 66 No. 34

**DOI:** 10.15585/mmwr.mm6638a7

**Published:** 2017-09-29

In the report, “Notes from the Field: Lead Poisoning in an Infant Associated with a Metal Bracelet — Connecticut, 2016” on page 916, the second paragraph should have read “The parents reported that the child intermittently wore a handmade “homeopathic magnetic hematite healing bracelet” that they had purchased from an artisan at a local fair (Figure). **The bracelet was described as "homeopathic," but homeopathic products are, by definition, regulated drugs and so nondrug items, such as bracelets, cannot be homeopathic. Cases of mislabeled products, especially among homemade items, should raise suspicion for consumers and health care professionals.** The child wore the bracelet for teething related discomfort and was sometimes noted to chew on it. Small spacer beads from the bracelet tested at the Manchester Health Department were positive for lead (17,000 ppm). No identifying marks indicating metal content or manufacturer were found on the bead. The vendor records were not available, and the bracelet maker could not be located.”

